# Diamine oxidase knockout mice are not hypersensitive to orally or subcutaneously administered histamine

**DOI:** 10.1007/s00011-022-01558-2

**Published:** 2022-03-18

**Authors:** Matthias Karer, Marlene Rager-Resch, Teresa Haider, Karin Petroczi, Elisabeth Gludovacz, Nicole Borth, Bernd Jilma, Thomas Boehm

**Affiliations:** 1 Department of Clinical Pharmacology, Medical University Vienna, Waehringer Guertel 18-20, 1090 Vienna, Austria; 2 Department of Neurophysiology, Center for Brain Research, Medical University Vienna, Vienna, Austria; 3 Department of Biotechnology, University of Natural Resources and Life Sciences, Vienna, Austria

**Keywords:** Amine oxidase (copper-containing), Histamine *N*-methyltransferase, Acute kidney injury, Metabolism, Tacrine, Metoprine, Body temperature

## Abstract

**Objective:**

To evaluate the contribution of endogenous diamine oxidase (DAO) in the inactivation of exogenous histamine, to find a mouse strain with increased histamine sensitivity and to test the efficacy of rhDAO in a histamine challenge model.

**Methods:**

Diamine oxidase knockout (KO) mice were challenged with orally and subcutaneously administered histamine in combination with the β-adrenergic blocker propranolol, with the two histamine-*N*-methyltransferase (HNMT) inhibitors metoprine and tacrine, with folic acid to mimic acute kidney injury and treated with recombinant human DAO. Core body temperature was measured using a subcutaneously implanted microchip and histamine plasma levels were quantified using a homogeneous time resolved fluorescence assay.

**Results:**

Core body temperature and plasma histamine levels were not significantly different between wild type (WT) and DAO KO mice after oral and subcutaneous histamine challenge with and without acute kidney injury or administration of HNMT inhibitors. Treatment with recombinant human DAO reduced the mean area under the curve (AUC) for core body temperature loss by 63% (*p* = 0.002) and the clinical score by 88% (*p* < 0.001). The AUC of the histamine concentration was reduced by 81%.

**Conclusions:**

Inactivation of exogenous histamine is not driven by enzymatic degradation and kidney filtration. Treatment with recombinant human DAO strongly reduced histamine-induced core body temperature loss, histamine concentrations and prevented the development of severe clinical symptoms.

**Supplementary Information:**

The online version contains supplementary material available at 10.1007/s00011-022-01558-2.

## Introduction

More than 95% of all histamine in humans is stored in mast cells and basophils. The same is likely to be true for many mammals. In humans, mast cell densities are highest in the gastrointestinal tract, the skin and lungs [1] and consequently these organs show histamine-induced symptoms in diseases with clear involvement of mast cell activation. The local interstitial concentrations of histamine after acute degranulation can reach 10–1000 µMs [2, 3, see Online Resources]. In humans, similarly to dogs and pigs, normal plasma histamine concentrations are below 1 ng/ml (9 nM) and symptoms start to develop at a few nanograms per milliliter [4–7]. Significant hypotension with increased heart rate can be measured from 5 ng/ml and when levels rise above 10 ng/ml the development of bronchospasm, cardiac arrhythmias, severe hypotension and coronary spasm can lead to life-threatening multi-system dysfunction [8, 9]. Histamine is not only involved in vasodilation, vascular permeability increases, hypoxia and the development of vascular edema, but also demonstrates pro-inflammatory involvement influencing the adaptive immune system via recruitment, maturation and activation of immune effector cells. Additionally it plays a role in the innate immune system by interacting with dendritic cells, natural killer cells and granulocytes [10, 11].

Baseline histamine concentrations in mice and rats are between 20 and 100 ng/ml when measured using reliable methods and are therefore many times higher than those found in humans [6, 7]. It is not clear whether these high histamine levels play any physiological role. Rodents are notoriously resistant to histamine with lethal doses 50% (LD_50_) in different mouse strains of 3000–4000 and 400–500 mg/kg after oral and intravenous administration, respectively [12, 13]. The peak plasma histamine concentration after a bolus administration of 400 mg/kg in a 20 g mouse would be approximately 8 mg/ml assuming a plasma volume of 1 ml. When anaphylaxis was induced in humans via a controlled wasp sting challenge, histamine concentrations of 140 ng/ml were associated with severe life-threatening hypotension [14]. How is histamine metabolized and inactivated?

Histamine shows 13% mean plasma protein binding and is freely filtrated in the kidneys [15]. The glomerular filtration rate (GFR) can theoretically contribute about 15–20% to the half-life of 3–4 min found in healthy volunteers [5, see Online Resources]. At a normal GFR of 100 ml/min and a plasma volume of 3000 ml the half-life of histamine would be 20 min. In mice, a normal GFR of 10 µl/min/g would result in a histamine half-life of 3 min [16, see Online Resources]. Nevertheless, histamine shows a high extraction rate in the kidney, exceeding rates based on the GFR, and this extraction is ascribed to uptake and reabsorption into the proximal tubular cells via organic cation transporter 2 (OCT2), followed by enzymatic inactivation [17, see below]. In humans, less than 1% of the injected radioactive histamine was found in urine within the first 6 h [18]. The low rate of histamine excretion in humans, which is also seen in dogs and cats, has been confirmed by others [19]. In mice and rat tissues more than 50% of the injected radioactivity is found in the kidneys, and less than 2% as histamine 30 min after intravenous administration [20]. The kidneys were also the organ with the highest radioactivity after high dose histamine administration in rats [21]. The OCT2 transporter is highly expressed in human and rodent kidneys, and might be responsible for both the extraction of histamine from the plasma compartment and the reabsorption of histamine in the primary urine filtrate into proximal tubular cells [22, see below].

Rapid transport of extracellular histamine from the interstitial fluid after release from mast cells or from plasma to other compartments away from the endothelial cells would be another possibility to inactivate histamine. This might inhibit induction of severe hypotension and vascular leakage mediated via endothelial nitric oxide synthase (NOS) signaling and binding to histamine receptors [23–25]. However, no in vivo animal data studying the transport rates of histamine from the systemic circulation into endothelial or parenchymal cells are available. In several in vitro studies, histamine has been shown to be transported bidirectionally using the low-affinity, high capacity OCT2 and OCT3 [22, 26, 27]. Histamine is an excellent substrate for the rat OCT2 and OCT3 transporter, showing higher transport efficiencies compared to the equivalent human OCT proteins [26].

When low concentrations of radioactive histamine are used in mice, methylation via histamine-*N*-methyltransferase (HNMT) seems to be the major route of inactivation. Challenging mice with higher doses of histamine, however, shifts metabolism to imidazoleacetic acid (IMAA) and riboside conjugates, with only a small amount of methylated derivatives detected [28–30]. The fivefold increased baseline serum histamine concentrations in the HNMT knock-out mice support these data [31]. Imidazoleacetic acid is generated via histamine oxidation by diamine oxidase (DAO) releasing imidazoleacetaldehyde, which is converted to IMAA and riboside derivatives, mainly in the liver. Oxidation of histamine via DAO appears to play a greater role in histamine catabolism after oral challenge in mice, which is not surprising considering that in mice DAO expression is high only in the gastro-intestinal tract [30]. When an oral histamine challenge is performed in humans oxidative deamination via DAO is the dominant catabolic pathway with IMAA as the main urinary metabolite [18].

Diamine oxidase is a copper-containing amine oxidase and one of two enzymes capable of inactivating histamine [32]. In selected tissues, mainly the small intestine and the kidney proximal tubular cells, DAO is located in ill-defined intracellular granular structures and extracellularly bound to heparan sulfate proteoglycans, whereas HNMT is present only in the cytoplasm [33, 34]. The expression of HNMT is widespread throughout the body, with higher levels found in the central nervous system, bladder, heart, kidneys, liver, lung and in adipose tissue.

After DAO inhibition using aminoguanidine, sheep showed extensive clinical symptoms of histamine toxicity after oral histamine challenge compared to controls without aminoguanidine pre-treatment [35]. A similar experiment in pigs resulted in severe morbidity and mortality in animals pre-treated with aminoguanidine and subsequently challenged with oral histamine [36]. Median plasma histamine concentrations increased 20-fold in pigs with DAO inhibition. Treatment of rats with aminoguanidine followed by oral histamine challenge increased urinary IMAA and reduced histamine concentration by approximately fivefold [37]. These data indicated that DAO plays a crucial role in the degradation of exogenous orally administered histamine.

In mice, aminoguanidine treatment strongly increased histamine concentrations in the intestine after intravenous histamine challenge [30]. However, aminoguanidine is not a specific DAO inhibitor, but blocks also all three NOS enzymes and appears to block transport of blood histamine into tissues [38, 39]. Administration of burimamide, a histamine receptor 2 antagonist, showed detrimental effects with increased mortality in a circulatory shock model in rats. However, at the same time it was published that burimamide is a potent DAO inhibitor [40, 41]. In dogs, burimamide caused a 17-fold increased mean plasma histamine concentration and a strong reduction in the mean arterial pressure [42]. The effect was believed to be due to possible mast cell activation.

Similarly, the role of HNMT was studied using methyl-histamine as an HNMT inhibitor, but methyl-histamine is also an excellent substrate for DAO [43]. Amodiaquine and quinacrine are potent HNMT inhibitors [44–46] but also inhibit DAO with an inhibitory concentration 50% (IC_50_) of approximately 500 nM [47, unpublished data]. Therefore, data derived using inhibitors, which are often used at high concentrations, must be interpreted with caution, because known and unknown off-target effects are likely and can significantly distort the physiological relevance of in vivo studies.

Metabolic studies might provide some indication of the importance of the two enzymes in the degradation of histamine, but catabolism of histamine could be decoupled from physiological or pathophysiological effects. The compartmental histamine concentrations could be critical and metabolism might be downstream.

We therefore decided to use the DAO knock-out (KO) mouse to study the role of DAO in the degradation of exogenous histamine. A second key aim was to develop a mouse model with increased histamine sensitivity to enable us to better study the role of histamine in various genetic mutant strains and to test the efficacy of recombinant human DAO in a histamine challenge model.

## Material and methods

### Animal models

Experiments were performed using 10–18-weeks-old C57BL6/J Aoc1^tm1b(EUCOMM)Hmgu^ (DAO) KO mice and wild type (WT) littermates. Heterozygous embryos were provided by the European Mouse Mutant Archive, Munich, Germany and implanted into pseudopregnant C57BL6/N mice. Heterozygous C57BL6/J offspring mice were confirmed using PCR (see Online Resources) and further used to breed DAO KO mice at the Division of Biomedical Research, Medical University of Vienna, in accordance with the animal protocol GZ 66.009/0160-WF/V/3b/2016. All animal experiments were conducted according to protocol GZ 66.009/0258-V/3b/2019. Experimental protocols were approved by the Austrian Ministry of Education, Science and Research. Animals were kept at a 12:12 h day–night cycle at 22 °C with water and food ad libitum. For non-invasive temperature measurements a transponder (IPTT-300, BioMedic Data Systems Inc., USA) was implanted subcutaneously 2 weeks prior to the experiment using short isoflurane anesthesia. The transponder measures the temperature three times within one second and the mean of these measurements is recorded from the outside of the cage using a reader. This mean value is then used for further calculations. These subcutaneous transponders are used to avoid excessive manipulation of animals and to prevent injury from repeated insertion of rectal temperature probes [48]. In several animal species including rodents histamine administration has been shown to lower body temperature and is considered the state of the art readout for the effects of histamine [49, 50]. During the observational period, clinical symptoms were evaluated by an experienced veterinarian according to a published hypersensitivity score [51]. The score ranged from 0 (no symptoms) to 1 (rubbing and scratching of head and nose), 2 (reduced activity with increased respiratory rate and/or reduced activity, puffiness around mouth and eyes), 3 (labored respiration, cyanosis around tail and mouth, wheezing) and 4 (no activity after prodding or tremor and convulsion). A score of 5 denoted death. All challenge experiments were started between 9:00 and 11:00 am to avoid time-of-day-dependent variations [52].

### General experimental setup

All substances were applied in a volume of 5 ml/kg. Propranolol (P0884, Sigma-Aldrich, Austria) was dissolved in saline and applied intraperitoneally (i.p.) at a concentration of 2 mg/kg 20 min prior to histamine challenge to increase sensitivity for histamine [53]. Histamine dihydrochloride (H7250, Sigma-Aldrich, Austria) was dissolved in double distilled (dd) H_2_O and further diluted in saline. All stated histamine concentrations refer to the histamine base (111.15 Dalton).

#### Oral and subcutaneous administration of histamine

Mice were fasted for 60 min in total. After 40 min of fasting, propranolol was administered and 20 min later histamine was applied at a concentration of 30 mg/kg per os (p.o.) using oral gavage. For the subcutaneous (s.c.) challenge model mice either received histamine at a concentration of 50 mg/kg without propranolol or 5 mg/kg with propranolol. For the determination of plasma histamine concentrations a subset of mice was anesthetized at different time points after histamine challenge using 10 mg/kg xylazine and 100 mg/kg ketamine. Citrate plasma was collected from anaesthetized mice using cardiac puncture. One to five mice were used per time point and genotype.

#### Concomitant histamine-*N*-methyltransferase (HNMT) inhibition

Metoprine (M338835, Toronto Research Chemicals, Canada) was dissolved in 10% lactic acid (L1875, Sigma-Aldrich, Austria), further diluted in saline and administered i.p. at 3 mg/kg 1 h prior to challenge with 5 mg/kg histamine. Tacrine (A79922, Sigma-Aldrich, Austria) was dissolved in ddH2O, further diluted in saline and subsequently 10 mg/kg were applied i.p. 1 h prior to 25 mg/kg histamine s.c. and 2 mg/kg propranolol. A concentration of 2 mg/kg Tacrine i.p. was used in combination with 30 mg/kg histamine p.o. combined with 2 mg/kg propranolol.

#### Induction of acute kidney injury (AKI) before histamine challenge

Folic acid (F7876, Sigma-Aldrich, Austria) was reconstituted in ddH2O, further diluted in saline and applied i.p. at a concentration of 100 mg/kg 48 h prior to challenge with 5 mg/kg s.c. histamine and 2 mg/kg propranolol. The degree of acute kidney injury was estimated using plasma creatinine values. As cut-off for inclusion a creatinine value of at least threefold above the mean baseline value was used as described [54]. Baseline plasma creatinine concentrations of 0.11 and 0.17 mg/dl were measured in two mice and therefore an inclusion cut-off of > 42 mg/dl was selected. Citrate plasma drawn via cardiac puncture was used to measure creatinine by means of a Cobas analyzer (Cobas C311 analyzer, Roche, Switzerland). To determine plasma histamine concentrations at different time points during s.c. histamine challenge with propranolol in acute kidney injury, two to four mice per time point were anesthetized and citrate plasma was collected by heart-puncture.

#### DAO rescue after histamine administration

Recombinant human (rh)DAO with a mutated heparin-binding motif (described in Gludovacz et al. [55]) was applied intravenously (i.v.) at a concentration of 4 mg/kg 40 min prior to application of 2 mg/kg propranolol and 60 min prior to challenge with 5 mg/kg s.c. histamine.

For determination of histamine and DAO concentrations in plasma, mice were treated with either 4 mg/kg DAO or buffer i.v. 60 min prior to challenge with 5 mg/kg s.c. histamine combined with 2 mg/kg propranolol. The mice were anesthetized at different time points. Citrate plasma was collected using cardiac puncture from two to three mice per time point. Blood was collected in 3.8% sodium citrate and one part was immediately mixed with diminazene-aceturate (D7770, Sigma-Aldrich, Austria) resulting in a final concentration of 10 µM to inhibit histamine degradation by rhDAO.

### Gene expression analysis

Tissue samples were shock-frozen in liquid nitrogen and total RNA was obtained using the FavorPrep Tissue Total RNA Kit (FATRK001, Favorgen, Taiwan) after tissue homogenization using lysing tubes (Lysing Matrix E, MP Biomedicals, Germany) on a Precellys 24 (Bertin Instruments, France). Reverse transcription was performed using the OneScript Plus cDNA synthesis kit (G236, ABM Good, Canada). For quantitative PCR BrightGreen Express 2× Mastermix (MasterMix-EL, ABM Good, Canada) was used. Exon spanning primers for DAO, HNMT and histidine decarboxylase (HDC) were designed using Primer3 software (Online Resource Table 1). The housekeeping gene RPLP0 was used for normalization [56].

### Western blot

For western blot analysis, frozen tissue samples were lysed in 20 mM K-phosphate buffer (pH 7.2) using lysing tubes (Lysing Matrix E tubes, MP Biomedicals, Germany) on a Precellys 24 (Bertin Instruments, France). Total protein concentration was determined using the QuantiPro BCA Assay Kit (QPBCA-1KT, Sigma, Austria). For Polyacrylamide gel electrophoresis 40 µg total protein and 40 ng recombinant murine DAO (provided by EG, University of Natural Resources and Life Sciences, Vienna, Austria, using methods described in [57]) were separated using a 12% Tris–glycine gel (4561043, Bio-Rad, USA). A monoclonal ABP1 antibody (sc-515908, Santa Cruz, USA) was used for DAO detection at a concentration of 0.4 µg/ml, and a monoclonal GAPDH antibody (2118, Cell Signal Technology, USA) was used as a loading control at a dilution of 1:2000. The monoclonal anti-mouse IgG-HRP antibody (A2554, Sigma Aldrich, Austria) and the anti-rabbit IgG-HRP antibody (A0545, Sigma Aldrich, Austria) were used as detection antibodies at a dilution of 1:40,000. Images were acquired using Clarity Max Western ECL Substrate (1705062, Bio-Rad, USA) on a ChemiDoc Imaging System (17001401, Bio-Rad, USA).

### DAO activity measurement

Diamine oxidase activity of different tissue homogenates and inhibition by metoprine and tacrine were measured as described [58]. Frozen tissue samples were lysed in 20 mM K-phosphate buffer (pH 7.2) using lysing tubes on a Precellys 24. Total protein concentration was determined using the QuantiPro BCA Assay Kit and 500 µg total protein extracts of different tissues were incubated for 120 min with ortho-aminobenzaldehyde (oABA) and either ddH2O or 200 µM cadaverine (CAD). Delta-1-piperideine, the autocyclization product of CAD after deamination by DAO, condensates with oABA forming a fluorophore, which can be measured at EX440/30 and EM620/40 nm. For determination of DAO inhibition, rhDAO was preincubated with metoprine and tacrine at different concentrations for 30 min and measured as described above.

For determination of DAO activity, tissue homogenates with a protein concentration of 200 µg/ml were mixed with HRP (final concentration 1.2 µg/ml, P6782, Sigma-Aldrich, Austria) and aminoguanidine (final concentration 10 µM, 396494, Sigma-Aldrich, Austria) or K-phosphate buffer (pH 7.2) were added and incubated for 15 min at 37 °C. Amplex red™ (final concentration 100 µM, A12222, Thermo Scientific, USA) was added and reactions were started via the addition of 200 µM final putrescine concentration (51799, Sigma-Aldrich, Austria). Potassium phosphate buffer (pH 7.2) was used as a negative control. Samples were incubated at 37 °C and measured every 10 min for 120 min using EX550 and EM590 nm. The DAO specific signal was calculated by subtracting samples with aminoguanidine, a potent and irreversible DAO inhibitor, from samples with K-phosphate buffer.

For plasma DAO activity measurements in mice receiving i.v. rhDAO a hybrid assay using a monoclonal antibody from the hybridoma cell line clone anti-DAO 8/119 provided by Prof. Quaroni (Cornell University, Ithaca, NY), and Amplex red™ was used. High-protein binding black fluorescence plates (475,515, Thermo Scientific Nunc, Denmark) were coated with 100 µl of 5 µg/ml anti-DAO 8/119 in 50 mM carbonate-bicarbonate buffer (C3041, Sigma-Aldrich, Austria), incubated overnight at 4 °C and subsequently blocked with 120 µl 1% BSA (A4503, Sigma-Aldrich, Austria) for 50 min at room temperature. After blocking 100 µl of plasma samples and standards, previously diluted 1:10 in PBS, were added and incubated for 1 h at room temperature. Afterwards 90 µl horseradish peroxidase (final concentration 1.2 µg/ml) and Amplex red™ (final concentration 100 µM) in PBS with 0.1% BSA were added and reactions were started by adding 10 µl putrescine (final concentration 200 µM) or PBS. Fluorescence was measured at 37 °C every 5 min for 120 min using EX550 and EM590 nm. The washing solution was 0.1% Tween-20 (P1379, Sigma-Aldrich, Austria) in PBS. A standard curve of 3–30 ng/ml rhDAO was prepared in mouse plasma. All measurements were performed in duplicate.

### Histamine measurements

Citrate plasma containing 10 µM diminazene-aceturate (D7770, Sigma-Aldrich, Austria) was used to measure histamine concentrations using the histamine homogeneous time resolved fluorescence (HTRF) dynamic kit (62HTMDPET, Cisbio, France). The kit was used according to the instructions provided by the manufacturer. A histamine standard curve in pooled plasma of C57Bl/6J mice was used for quantification. All histamine concentrations refer to the histamine base and all measurements were performed in duplicate.

### Statistical analysis

Statistical analyses were performed using GraphPad Prism Version 8.4.0. (GraphPad Software Inc. San Diego). Statistical significance for differences in DAO activity in tissue homogenates was calculated using a repeated-measures ANOVA with Geisser–Greenhouse correction. The area under the curves (AUC) from individual core body temperature measurements and clinical scores during the course of an experiment were compared using two-sided, unpaired t-tests without Welch’s correction. Plasma histamine concentrations between groups were compared with a two-way ANOVA. For comparison of plasma histamine concentrations after s.c. challenge between mice with different genotypes, with and without acute kidney injury and receiving either DAO or buffer, data were grouped into intervals to account for missing values in individual subgroups (genotypes: 5, 10, 20, 30, 40, 45, 60 min; acute kidney injury: 0, 10–15, 20–30, 40–45 and 60 min; DAO: 0, 10, 30 and 45 min). A two-sided, unpaired *t* test was used to test for differences in plasma creatinine concentrations from mice treated with and without 100 mg/kg folic acid. Statistical significance was defined as *p* < 0.05 in all tests.

## Results

### Characterization of DAO knockout (KO) mice

**Fig. 1:**
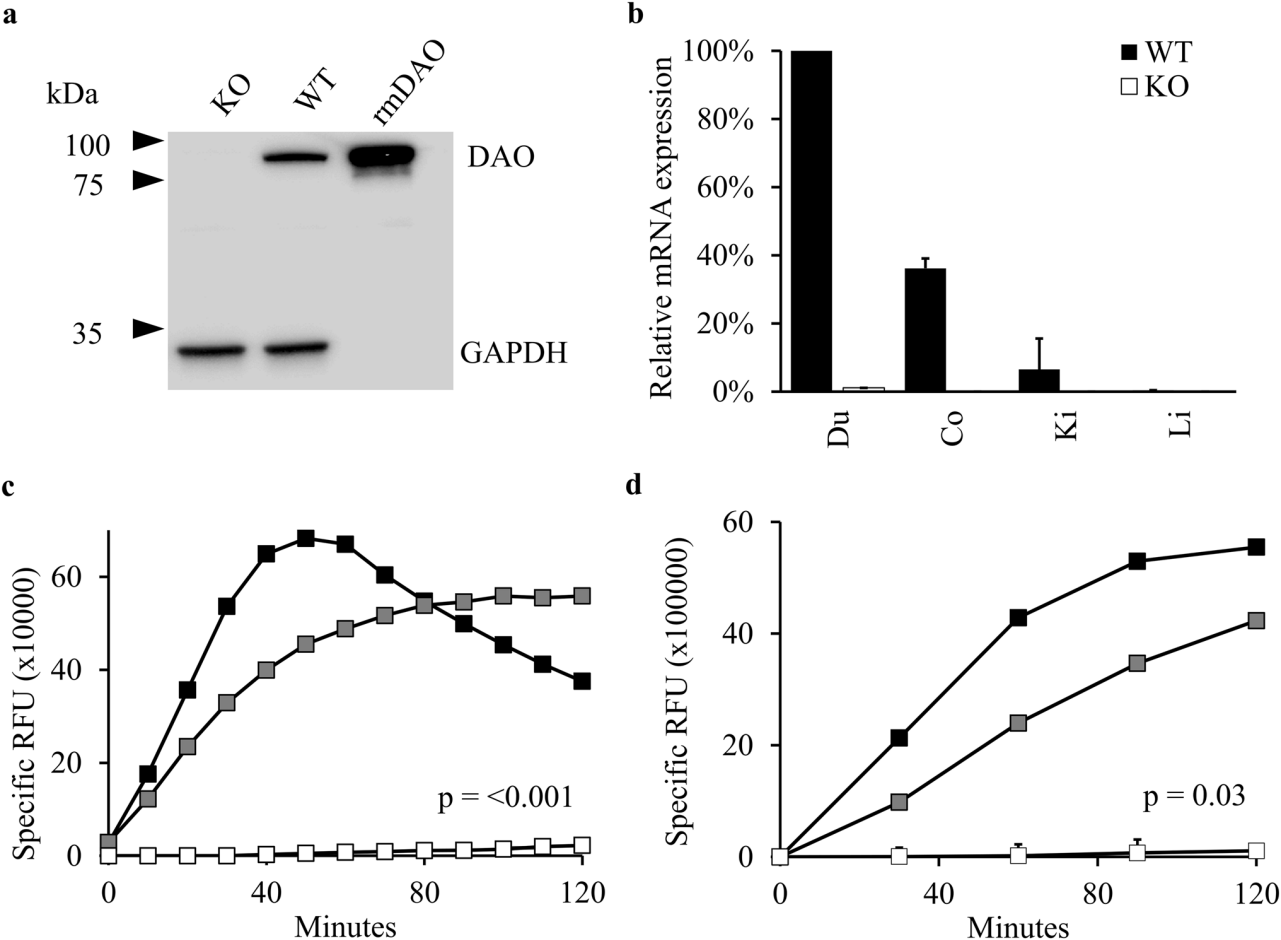
No DAO mRNA, antigen and enzymatic activity in DAO KO mice. **a** Western blot analysis of 40 µg tissue homogenates from duodenal samples of WT and DAO KO mice and 40 ng recombinant murine (rm)DAO. **b** For quantitative PCR of DAO mRNA expression data from duodenum (Du), colon (Co), kidney (Ki) and liver (Li) tissue homogenates of one DAO KO and WT mouse were normalized to Ribosomal Protein Large P0 (RpLp0) mRNA and the mean of duplicates with standard errors of the mean (SEM) expressed relative to WT duodenum samples. **c**, **d** DAO activity measurements of tissue homogenates from duodenum of one WT (filled black square), heterozygous (filled grey square) and DAO KO (open square) mouse using putrescine oxidation with horseradish peroxidase/hydrogen peroxide/Amplex red™ coupling (**c**) or using fluorophores generated after fusion of delta-1-piperideine (oxidation product of cadaverine) and ortho-aminobenzaldehyde (**d**) were significantly different in both independent assays (*p* ≤ 0.001 and *p* = 0.03 including all curves using repeated measures ANOVA with Geisser-Greenhouse correction). Results of DAO activity measurements in tissue extracts show the mean of duplicates with SEM of < 11,000 RFU for **c** and < 24,200 RFU for **d** for all time points. *RFU* relative fluorescence units

The absence of DAO protein and mRNA was confirmed by comparing duodenum homogenates from wildtype (WT) and KO mice using western blot and qPCR analysis (Fig. 1a, b). Correct antibody detection was verified by employing recombinant murine DAO. Quantitative PCR of DAO mRNA from several tissues showed a minimal or absent signal in the KO mouse (Fig. 1b, Online Resource Fig. 1). The relatively low DAO mRNA expression in the WT mouse kidney extract might be an underestimation since DAO expression is restricted to proximal tubular cells, but they constitute only a small percentage of kidney tissue. Expression of HNMT and HDC in WT and KO mice did not show significant differences (Online Resource Fig. 1).

Enzymatic activity measurements of DAO in WT, heterozygous and homozygous KO duodenum protein homogenates confirmed the absence of DAO activity in KO mice using two independent assays (Fig. 1c, d). Samples from heterozygous mice showed a 59% and 46% signal increase (slope) compared to WT mice within the first 30 min using an Amplex red™ (Fig. 1c) or ortho-aminobenzaldehyde-based assay (Fig. 1d) respectively.

### No differences in the reduction of body temperatures between DAO WT and KO mice after exogenous oral and subcutaneous histamine challenge

**Fig. 2:**
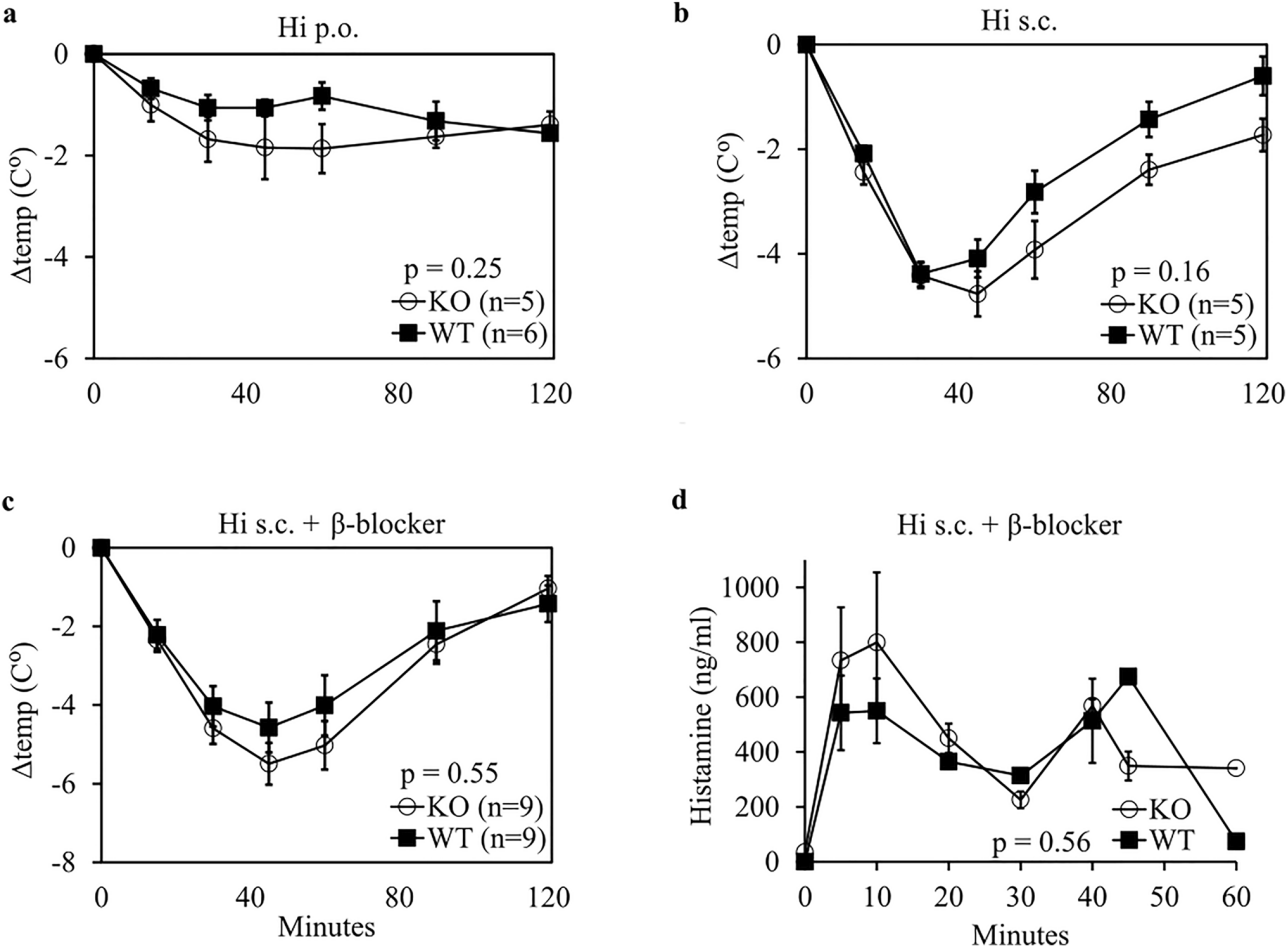
No difference in body temperature reduction between WT and DAO KO mice after oral or subcutaneous histamine challenge. Mice were either challenged with **a** 30 mg/kg histamine (Hi) oral (p.o.) alone, **b** 50 mg/kg Hi subcutaneous (s.c.) alone or **c** 5 mg/kg Hi s.c. in combination with 2 mg/kg propranolol (β-blocker) intraperitoneal (i.p.) 20 min prior to Hi. Differences in core body temperatures normalized to baseline are shown as mean ± SEM. *p* values were calculated using a two-sided, unpaired *t* test of individual area under the curves (AUCs). **d** Mean (± SEM) plasma Hi levels of mice at different time points challenged with 5 mg/kg Hi and 2 mg/kg propranolol. Plasma was collected via cardiac puncture using 3–7 mice per time point. The *p* value was calculated using a two-way ANOVA comparing Hi plasma concentrations of WT and DAO KO mice. *SEM* standard error of the mean

As DAO expression is high in the gastrointestinal tract, the largest phenotypical difference might be measured after oral histamine challenge. After administration of 30 mg/kg histamine p.o., we observed no relevant difference in the reduction of the core body temperature between DAO KO and WT mice (Fig. 2a). In the next series of experiments, histamine was injected s.c. at 50 mg/kg to induce a measurable clinical response, but no significant differences were observed between DAO WT and KO mice (Fig. 2b). The high histamine concentrations necessary to provoke a relevant temperature drop might have masked the involvement of enzymatic degradation. We added the β adrenergic blocker propranolol, because it has been shown to significantly increase histamine sensitivity [59]. We hypothesized that reduced histamine exposure might shift histamine inactivation/clearance to enzymatic pathways. The addition of propranolol resulted in the same temperature drop with ten-times less histamine. Nevertheless, no phenotypical differences were detected between the genotypes (*p* = 0.55) (Fig. 2c). The mean (SEM; *n* = 4) plasma histamine concentration was 34 (9.5) ng/ml at baseline. There were no significant differences between WT and DAO KO mice in plasma histamine concentrations after s.c. histamine challenge with propranolol pre-treatment (*F*(6, 21) = 0.83, *p* = 0.56). Mean (SEM) histamine concentrations were 799 (255) ng/ml in KO (*n* = 2) and 550 (117) ng/ml in WT mice (*n* = 4) (Fig. 2d) 10 min after challenge with 5 mg/kg histamine and 2 mg/kg propranolol.

### Histamine-*N*-methyltransferase (HNMT) inhibition does not significantly influence histamine sensitivity of DAO WT and KO mice

**Fig. 3:**
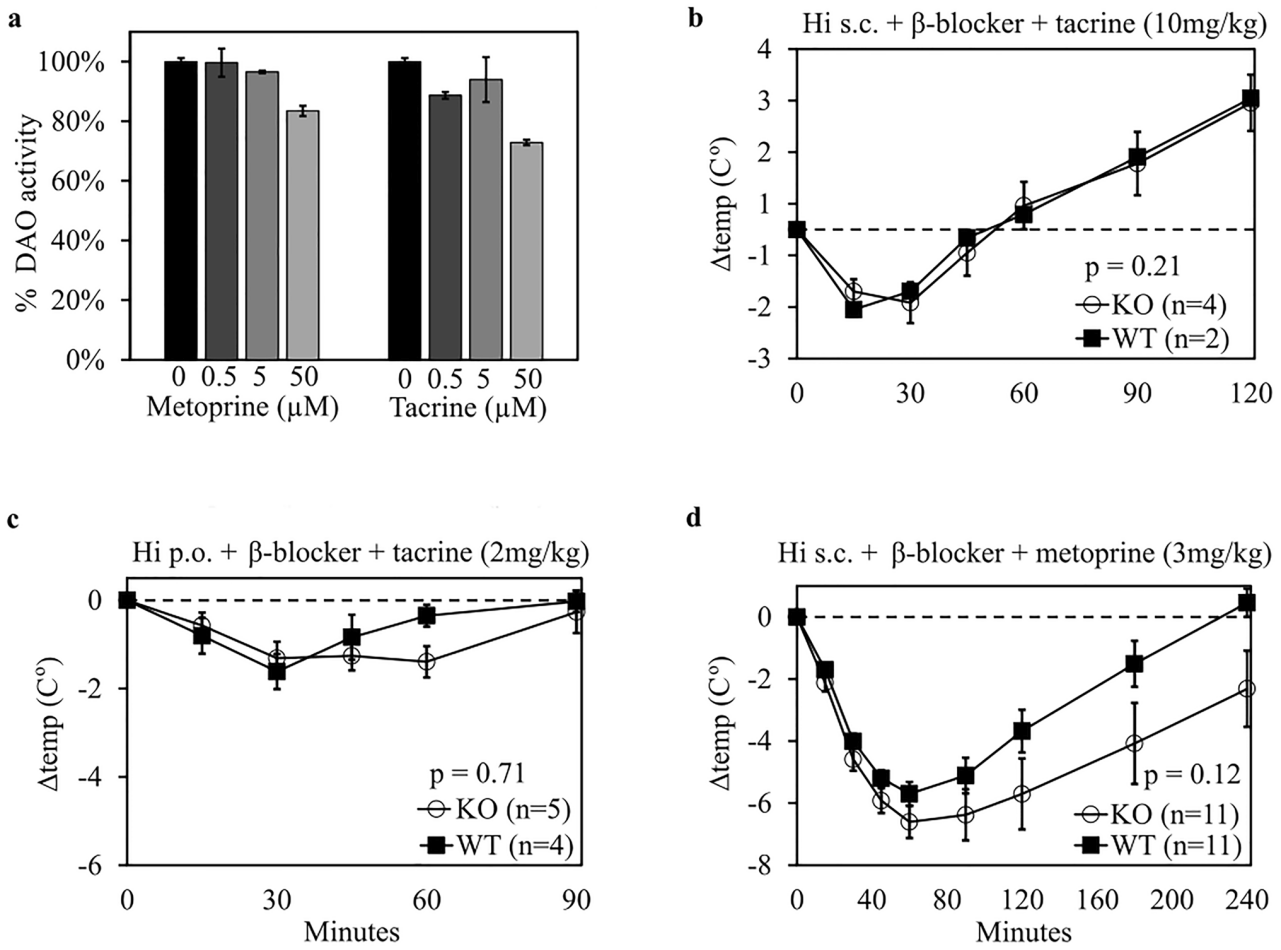
Inhibition of HNMT does not increase histamine sensitivity in mice. **a** Mean inhibition of recombinant human DAO (rhDAO) by metoprine and tacrine was measured using an activity assay based on the fusion of delta-1-piperideine (DAO oxidation product of cadaverine) with ortho-aminobenzaldehyde generating a fluorophore followed by fluorescence measurements. Means (± SEM) of duplicates are shown. **b** WT and DAO KO mice were treated with tacrine (10 mg/kg intraperitoneal, i.p.) 60 min before challenge with histamine (Hi, 25 mg/kg subcutaneous, s.c.) and the β-blocker propranolol (2 mg/kg i.p.). **c** Mice received tacrine (2 mg/kg i.p.) 60 min and propranolol (2 mg/kg i.p.) 20 min prior to Hi (30 mg/kg p.o.) challenge. **d** Metoprine (3 mg/kg i.p.) was administered 40 min before propranolol (2 mg/kg i.p.) and 60 min before Hi (5 mg/kg s.c.). Differences in core body temperature normalized to baseline are shown as mean (± SEM). *p* values were calculated using a two-sided, unpaired *t* test of individual area under the curves (AUCs)

The chemical structures of tacrine and metoprine, two potent HNMT inhibitors [45], indicate that they might not be potent DAO inhibitors. Diamine oxidase activity was minimally inhibited at 5 µM and was inhibited 20–30% at 50 µM (Fig. 3a). Blocking HNMT with relatively high concentrations of two independent inhibitors did not show a relevant difference between KO and WT mice after challenge with orally or subcutaneously administered histamine (Fig. 3b–d). Mice receiving 10 mg/kg (50 µmol/kg) tacrine showed extremely low body temperatures of < 34.4 °C compared to 37.0 (SD 0.87; *n* = 18) for untreated animals, reduced mobility and ataxia-like movements 1 hour after tacrine application prior to histamine challenge. Subsequent subcutaneous histamine challenge led to a mean drop of just − 1.3 °C after 30 min and a subsequent rise to + 2.5 °C after 120 min, possibly attributed to mice restoring initial body temperature (Fig. 3b). The subsequent challenges were performed with 2 mg/kg (10 µmol/kg) tacrine, which showed a minimal baseline temperature drop before histamine challenge, moderate reduced mobility and minimal ataxia-like movements after tacrine administration [60]. A trend towards increased temperature loss in DAO KO mice might be present after 3 mg/kg (11.1 µmol/kg) metoprine administration (*p* = 0.12).

### Acute kidney injury increased plasma histamine concentrations but did not affect histamine body temperature loss in DAO WT and KO mice

Baseline plasma creatinine concentrations of ≤ 0.17 mg/dl (*n* = 2) increased to 1.25 mg/dl (*n* = 19, SD 0.55) 48 h after 100 mg/kg folic acid treatment (*p* = 0.013, two-sided unpaired *t* test). This increase is congruent with published data [54]. Three mice with plasma creatinine levels below 0.45 mg/dl after folic acid treatment were excluded from the present study because of potentially insufficient kidney injury. According to the literature, serum creatinine levels start to increase, when the glomerular filtration rate (GFR) decreases by 30%. At creatinine concentrations > 0.45 mg/dl the GFR is likely < 4 µl/min/g compared to a normal baseline GFR of 11.5 µl/min/g. This corresponds to a 65% GFR reduction [16]. The acute kidney injury induced by folic acid is mainly caused by the formation of crystals in the tubular lumen, acute tubular necrosis with tubular dilatation and brush border loss [61].

**Fig. 4:**
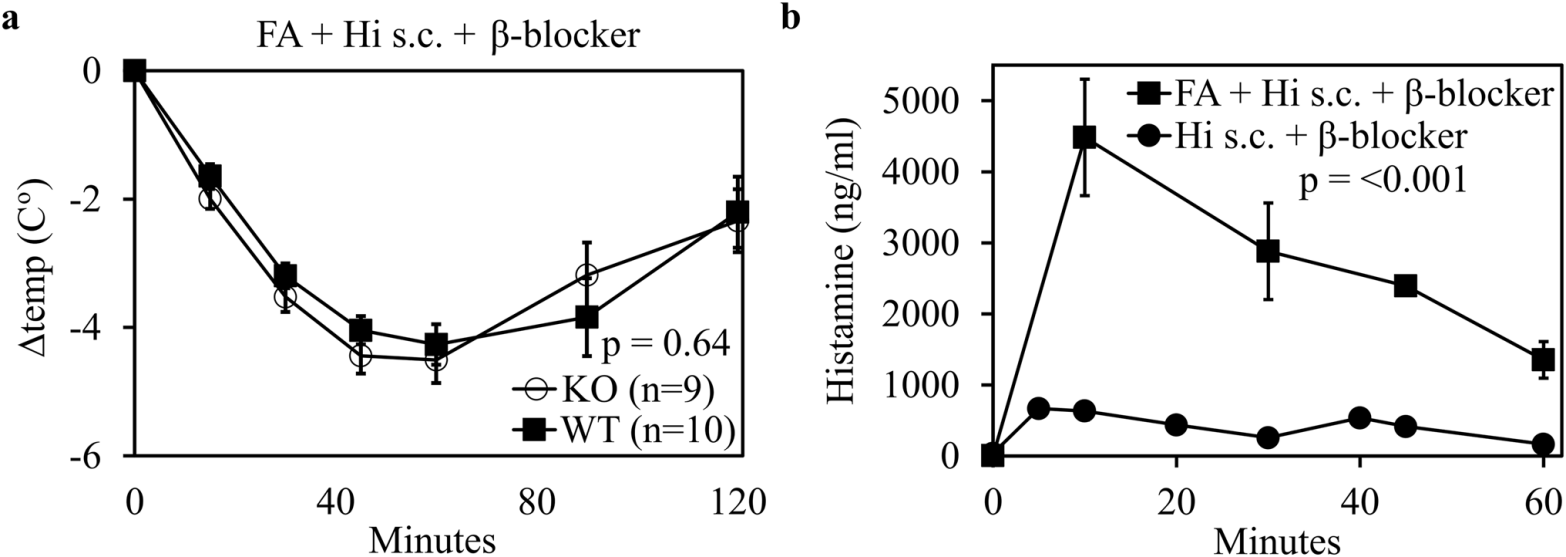
Acute kidney injury increased circulating histamine concentrations but did not influence the body temperature loss in WT and DAO-KO mice. **a** Mice received 100 mg/kg folic acid (FA) intraperitoneally (i.p.) leading to acute kidney injury within 48 h. These mice were also treated with the β-adrenergic blocker propranolol at 2 mg/kg i.p. 20 min prior to histamine (Hi) challenge (5 mg/kg s.c.). Differences of core body temperatures normalized to baseline are shown as mean ± SEM. *p* values were calculated using a two-sided unpaired *t* test of individual area under the curves (AUCs). **b** Plasma Hi levels of 2–4 mice with acute kidney injury induced by FA (100 mg/kg, i.p.) and challenged with Hi (5 mg/kg s.c.) and propranolol (2 mg/kg i.p.), were determined at different time points post Hi challenge. Plasma Hi concentrations measured in duplicate of FA-treated mice are shown as the pooled mean (± SEM) of DAO KO and WT mice. The *p* value was calculated using a two-way ANOVA comparing the mean of WT and DAO KO mice with acute kidney injury (filled square) to the mean of WT and DAO-KO mice with normal kidney function (filled circle) after challenge with Hi (5 mg/kg s.c.) and propranolol (2 mg/kg i.p.). The control mice data are identical to the data from Fig. 2d combining data from WT and KO mice. Folic acid-treated mice were only included in the analysis if creatinine levels increased at least threefold compared to baseline or > 0.45 mg/dl indicating a glomerular filtration rate reduction of at least 65%

No differences in the core body temperature reduction were observed between DAO KO and WT mice (*p* = 0.64) after acute kidney injury (Fig. 4a). Peak mean (SEM) plasma histamine levels increased from 633 (125) to 4483 (818) ng/ml or 7.1-fold in mice with acute kidney injury. A two-way ANOVA of the histamine concentrations resulted in a statistically highly significant difference between mice with impaired and normal kidney function (*F*(4, 44) = 23.2, *p* < 0.001) (Fig. 4b). Plasma histamine levels did not correlate with measured core body temperature changes.

### Recombinant human (rh) DAO protects mice from histamine-induced temperature loss and development of severe clinical symptoms

**Fig. 5:**
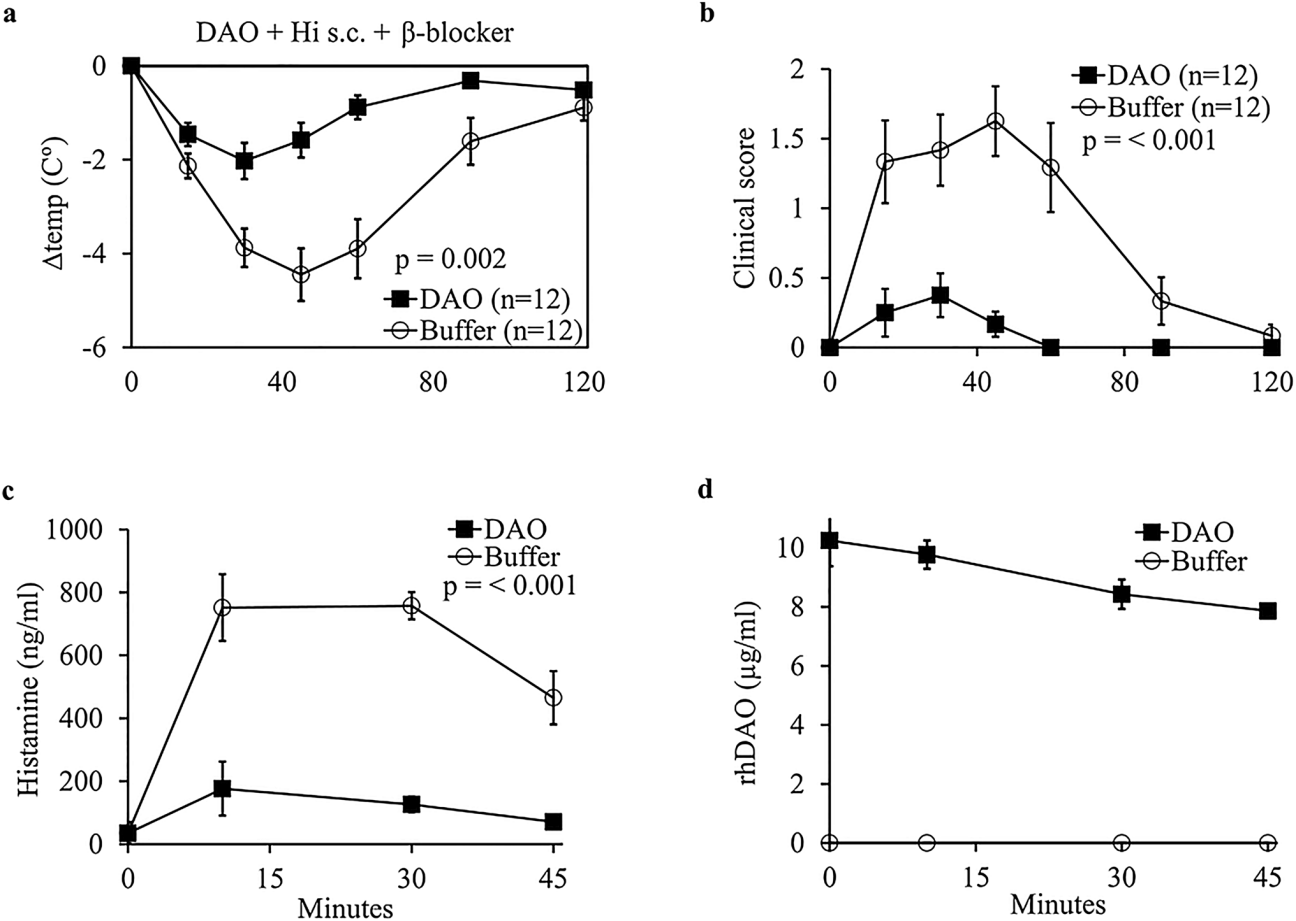
Recombinant human DAO protects mice from histamine-induced reduction of core body temperature and the development of severe symptoms. **a** Recombinant human (rh)DAO (4 mg/kg intravenous, i.v.) was applied 40 min before administration of propranolol (2 mg/kg intraperitoneal, i.p.) and 60 min prior to challenge with histamine (Hi, 5 mg/kg subcutaneous, s.c.). Differences in core body temperature normalized to baseline are shown as mean ± SEM. The *p* value was calculated using a two-sided unpaired *t* test of individual areas under the curve (AUCs). **b** Mean clinical score (± SEM) of mice receiving rhDAO (4 mg/kg i.v.) or buffer (i.v.) prior to propranolol (2 mg/kg i.p.) and Hi (5 mg/kg s.c.). The *p* value was calculated using a two-sided unpaired *t* test of individual AUCs. **c** Mean (± SEM) plasma Hi levels measured of *n* = 2–4 mice per time point receiving either DAO (4 mg/kg i.v.) or buffer (i.v.) 40 min prior to propranolol (2 mg/kg i.p.) and 60 min prior to challenge with Hi (5 mg/kg s.c.) are shown. A two-way ANOVA was used to compare Hi concentrations. **d** Mean (± SEM) plasma rhDAO concentrations at different time points (*n* = 3 for each time point) were calculated using a monoclonal anti-DAO antibody to immobilize DAO on a microtiter plate coupled with Amplex red™ DAO activity measurements using an rhDAO standard curve

One goal of challenging the DAO KO mouse with histamine was to find a mouse strain with increased histamine sensitivity. A further aim was to test the efficacy of rhDAO in different histamine-associated disease models. Because no difference between DAO KO and WT mice was observed, we tested histamine degradation capabilities of rhDAO in ten WT and two DAO KO mice. Mice were pretreated with rhDAO or buffer intravenously and subsequently challenged with s.c. histamine 1 h after DAO application. A heparin-binding motif mutated rhDAO-R568S/R571T variant with strongly reduced clearance was used at 4 mg/kg [55]. Mice receiving intravenous rhDAO showed a statistically highly significant lower peak mean (SEM) temperature loss of − 1.6 °C (0.4) after 45 min compared to buffer controls, which demonstrated a temperature loss of − 4.4 °C (0.6) with a *p* value of < 0.001 using an unpaired t-test for individual core body temperature reductions (Fig. 5a). The data correspond to an AUC reduction of the core body temperature loss of 63% after pretreatment with rhDAO (*p* = 0.002 using an unpaired *t* test of individual AUCs). During histamine challenge, mice showed various moderate to severe clinical symptoms including reduced movement and buccal swelling resulting in a statistically highly significantly increased mean clinical score compared to mice receiving i.v. rhDAO, who showed mild or indeed no clinical symptoms during the observation period (*p* < 0.001, Fig. 5b).

Histamine concentrations in mice receiving prophylactic DAO were statistically highly significantly different compared to buffer control animals (ANOVA, *F*(3, 17) = 18.69, *p* < 0.001, *n* = 2–4 per group, Fig. 5c). The histamine AUC decreased by 81%. The active plasma rhDAO concentrations were estimated using an activity assay using a standard curve with rhDAO from the same batch (Fig. 5d). The monoclonal anti-human DAO antibody does not recognize mouse DAO. We therefore do not know endogenous mouse DAO concentrations.

## Discussion

When approved medications are administered to humans histamine-like adverse events are seen quite frequently. However, it is rare that inhibition of DAO and HNMT are considered as contributing factors. The in vivo relevance of both enzymes in the degradation of histamine is not yet understood, and simple extrapolation from in vitro enzyme inhibition data to the in vivo situation is certainly not justified.

High dose nafamostat administration during cardiopulmonary bypass operations showed elevated histamine concentrations, and the authors suggested nafamostat-mediated DAO inhibition as a possible cause [62]. Bisantrene was approved for the treatment of acute myeloid leukemia in 1988 and might soon be tested again in clinical trials for acute myeloid leukemia, myelodysplastic syndrome and breast cancer [63]. Hypersensitivity reactions were a common adverse event in clinical studies [64]. Diamine oxidase inhibition could be suspected because bisantrene possesses two terminal dihydro-imidazole moieties with two internal hydrazine groups, both chemical structures present in highly potent and partially irreversible DAO inhibitors like aminoguanidine, diminazene aceturate and imidocarb [32, 47].

Here, we have shown that DAO KO mice have no remaining DAO mRNA expression or activity. We then challenged these mice with histamine to develop a model in which suspected DAO inhibitors could be tested. If we had found a strong difference between WT and DAO KO mice, treatment with potent DAO inhibitors in WT mice should result in a DAO KO comparable phenotype. Nevertheless, DAO KO mice behaved similarly to WT mice after oral or subcutaneous histamine challenge in our experimental settings. Based on large animal studies in pigs and sheep using the potent and irreversible, albeit not specific, DAO inhibitor aminoguanidine deletion of DAO protein expression was expected, at least after oral challenge, to show an adequate phenotype. The highest mRNA and DAO activity levels are localized in the small intestine of mice. Nevertheless, pigs and sheep show low baseline histamine levels compared to humans and might be significantly more histamine sensitive when compared to mice or rodents. In pigs, 2.4 mg/kg orally administered histamine caused severe morbidity and a 20% death rate after aminoguanidine treatment [36]. The LD_50_ in mice using oral histamine challenge is at least 1000-times higher [12, 13]. Thirty mg/kg of histamine administered orally to DAO KO mice showed only a mild to moderate phenotype with no difference to the DAO WT animals. Off-target effects of high dose aminoguanidine treatment in pigs and sheep might also have contributed to severe morbidity and mortality. Aminoguanidine inhibited in vitro bovine HNMT only 24% at 100 µM, but in rat intestinal DAO an inhibition of 78% was noted at 1 µM [47]. It is not likely that aminoguanidine blocked both enzymes, DAO and HNTM, in the pig or sheep studies.

In mice the catabolism of histamine is very rapid and together with the well documented histamine resistance the influence of enzyme absenteeism, certainly from DAO, was not discernable. Even 2.5 min after intravenous injection of radioactive histamine less than 5% of the injected radioactivity is present as histamine in the blood compartment [30, 65]. This fast removal cannot be explained via kidney filtration. The C57BL6 strain showed a 4 °C temperature drop after subcutaneous administration of 5 mg/kg or 100 µg histamine per 20 g mouse or a plasma concentration of 96 µg/ml, but peak histamine concentrations were “only” 800 ng/ml after 10 min. Most of the histamine is rapidly removed from the plasma compartment and transferred into tissues, predominantly into the liver, the kidneys and the gastrointestinal tract, possibly using mainly the transporters OCT2 and OCT3. In these three tissue compartments histamine might not be able to readily activate enough histamine receptors to induce systemic symptoms. This rapid transfer of histamine from the subcutaneous and consequently intravenous compartment into cells or the interstitial fluid might partially explain the robust histamine resistance in rodents. Only very high plasma concentrations of histamine can sufficiently activate histamine receptors followed by nitric oxide synthesis and release, finally leading to vascular permeability increases and consequently to a temperature drop. At these high concentrations the rapid transport of histamine might be saturated.

After stimulation of endothelial cells with bradykinin or histamine the release of nitric oxide can be measured in approximately 100 ms [66]. Therefore the development of vascular leakage and permeability increases after histamine administration are also likely to be rapid. Subcutaneously administered histamine peaked after 5 min in plasma (as demonstrated in this study) and it might be reasonable to assume that plasma histamine is rapidly able to increase endothelial cell permeability, allowing transfer of histamine into all tissues and not just tissues with high OCT2 and OCT3 expression. This hypothesis might be tested by performing oral and subcutaneous histamine challenges in OCT2, OCT3 and double OCT2/3 KO mice. If this hypothesis is correct, OCT2 and OCT3 KO mice should be equally histamine sensitive compared to WT mice, because histamine is rapidly transferred into all tissues irrespective of the OCT2/3 transporters. If OCT2 and OCT3 transfer rates are “dominant” and if rapid transport of histamine via OCT2 and OCT3 can block symptom development, the transporter KO mice might demonstrate significantly increased plasma histamine concentrations and sensitivity after exogenous histamine challenge.

In rodent in vivo studies another possible route of rapid histamine inactivation could be the incorporation of histamine into proteins [67]. Post-translational histaminylation of the GTPases Cdc42, Gαo1 and Gαq was shown to occur in cell culture experiments and could also contribute to the in vivo clearance of plasma histamine [68]. Interestingly, Gαo1 and Gαq seem to be involved in vesicular histamine transport and became constitutively active after histaminylation.

When we pretreated mice with the potent HNMT inhibitors tacrine and metoprine, both showed minimal DAO inhibitory activity at 50 µM in vitro, a trend for slower recovery to baseline temperature values in the DAO KO mice might have been present in the metoprine-treated mice. Tacrine at 10 mg/kg caused significant adverse effects, but even when administered at 2 mg/kg with minimal side effects no difference in the temperature drop was seen between DAO WT and KO mice. Metoprine did not change the slope in temperature loss for the first 60 min until the peak was reached, but might have prolonged recovery in the DAO KO mouse. This might indicate that DAO plays some role in the final inactivation of histamine, but not during the early and peak phase. A comparison of the temperature drop after an equal histamine challenge with or without metoprine treatment showed statistically highly significant differences in the AUC data, indicating that metoprine, or possibly HNMT activity, might influence histamine sensitivity, but again only in the last phase and not during the first 60 min (Online Resources Fig. 3). We cannot exclude off-target effects of metoprine. A double DAO/HNMT KO mouse, if viable, might provide a more definite answer about the involvement of DAO/HNMT in histamine sensitivity. Did we significantly block HNMT activity in mice treated with tacrine or metoprine, thus generating a “double” DAO/HNMT KO mouse?

In rats, tacrine blood concentrations were approximately 9 µM (1.8 µg/ml), with levels almost ten times higher noted in the kidneys, liver and pancreas 30 min after intravenous administration of a 5 mg/kg dose [69]. In several brain structures, tacrine concentrations reached 25 µM, or approximately threefold higher compared to plasma, possibly explaining the adverse events we saw with 10 mg/kg tacrine. The lipophilic nature of tacrine with a log*D* of 1.25 should readily allow inhibition of HNMT in the cytoplasm of many tissue cells. The inhibition constant *K*_i_ and IC_50_ for rat HNMT was described to be 35 and 74 nM, respectively [70]. Horton et al. [45] published a *K*_i_ of 38 nM using recombinant human HNMT. In brain extracts, rat HNMT was inhibited approximately 80% when administered at a dose of 5 and 10 mg/kg tacrine [70]. Tacrine should have inhibited mouse HNMT more than 70% in our experiments.

Metoprine with a log*D* of 2.0 is more lipophilic compared to tacrine and shows a long half-life of 19 h in mice [71]. When applying 1 mg/kg in rats, concentrations in brain, lung and pancreas were 7-, 31- and 5-fold respectively above plasma levels after 5 h. High tissue concentrations were also described in a patient 7 days after the last metoprine dosing [71]. The inhibition constant *K*_i_ for bovine, bovine and human HNMT were 58, 100 and 91 nM, respectively [44, 45, 72]. In rats, metoprine inhibited HNMT activity from whole brain extracts 70% at 3 mg/kg and 75% between 5 and 40 mg/kg with no dose-dependent response [73]. Considering that brain concentrations are often lower compared to other tissues, HNMT activity after administration of metoprine at 3 mg/kg was likely to be more than 70% inhibited in our experiments.

Folic acid-induced acute kidney injury increased the AUC of circulating histamine concentrations sevenfold, but did not lead to a separation of the temperature loss curves between DAO WT and DAO KO mice. The temperature loss curves after subcutaneous histamine and β-blocker administration are not significantly different between folic acid-treated and control mice (Online Resources Fig. 4). As expected from metabolic studies, this indicates not only that the kidneys are a major histamine extractor, but also that circulating plasma histamine might not play a dominant role in the induction of the temperature loss. Rapid wide-spread tissue uptake of administered histamine might be more important compared to kidney-mediated histamine extraction. These considerations assume that folic acid did not significantly influence temperature control mechanisms masking any effects of higher circulating histamine concentrations.

The presence of 10 µg/ml (60 nM) rhDAO in plasma was able to rapidly remove approximately 80% of the high histamine concentrations and to concomitantly reduce symptomatology from a severe (≥ 4 °C temperature loss) to a mild or moderate (≤ 2 °C) course. This is the first description of the therapeutic potential of a heparin binding motif-mutated rhDAO variant using an exogenous high concentration histamine challenge model. Porcine kidney DAO was beneficial in the inhibition of small intestinal and renal post-ischemic reperfusion injury in rats [74, 75]. Recombinant hDAO might be tested in other animal models with a suspected contribution of mast cell-derived or freshly synthesized histamine. For longer treatment periods, recombinant mouse DAO might be used to avoid antibody generation and possible interference.

In conclusion, DAO KO mice were essentially indistinguishable from WT mice using exogenous oral and subcutaneous histamine challenges. The involvement of HNMT in the inactivation of histamine leading to reduced symptomatology is moderate at best, but results from pharmacological inhibition might be considered preliminary. Data using inhibition of HNMT with metoprine showed a trend towards the involvement of endogenous DAO in histamine inactivation. The kidneys are clearly involved in the rapid extraction of histamine from the circulation, but the sevenfold elevated histamine concentrations did not translate into an exaggerated phenotype measured using central temperature loss as phenotypical readout. The use of recombinant human or mouse DAO might support or help dismiss the involvement of histamine in various animal models with suspected mast cell degranulation accompanied by rapid and massive histamine release, or with enhanced induction of the histidine decarboxylase enzyme followed by release of freshly synthesized histamine over hours. Breeding a true double KO mouse with inactive copies of both the DAO and the HNMT gene, if viable, might be worth studying. Single KO mice of either DAO or HNMT do not show obvious phenotypes.[unpublished data, 31] Similarly, testing the involvement of the two main histamine transporters, OCT2 and OCT3, in histamine-induced phenotypical alterations might allow us to gain a better understanding of the mechanisms behind the development of histamine-mediated symptoms in rodents and consequently potentially also in humans. Despite significant research efforts for more than 100 years since its discovery we still have some way to go before we gain a true understanding of histamine catabolism.

## Supplementary Information

Below is the link to the electronic supplementary material.

Supplementary file1 (DOCX 2613 KB)
