# Supplementary material for: Diamine oxidase knockout mice are not hypersensitive to orally or subcutaneously administered histamine
Source: Inflamm Res. 2022 Mar 18;71(4):497–511. doi: 10.1007/s00011-022-01558-2 (PMC8989821; doi:10.1007/s00011-022-01558-2)
Supplement: Supplementary file 1 — Supplementary file1 (DOCX 2613 KB) [file 11_2022_1558_MOESM1_ESM.docx]

**Supplement to**

**Diamine oxidase knock out mice are not hypersensitive to orally or subcutaneously administered histamine**

Matthias Karer^1^, Marlene Rager-Resch^1^, Teresa Haider^2^, Karin Petroczi^1^, Elisabeth Gludovacz^3^, Nicole Borth^3^, Bernd Jilma^1^, Thomas Boehm^1^*

^1^ Department of Clinical Pharmacology, Medical University Vienna, Vienna, Austria

^2^ Department of Neurophysiology, Center for Brain Research, Medical University Vienna, Vienna, Austria

^3^ Department of Biotechnology, University of Natural Resources and Life Sciences, Vienna, Austria

*Corresponding author

Thomas Boehm

Department of Clinical Pharmacology

Medical University Vienna

Waehringer Guertel 18-20, 1090 Vienna, Austria

Tel.: +43 1 40400 49580

Fax.: +43 40400 29980

Email: thomas.boehm@meduniwien.ac.at

Orcid: 0000-0002-8294-0797

**Online Resources Table 1** Primers for qPCR and Genotyping

| **Used as** | **Name** | **Sequence (5‘-3‘)** | **Product / Name** |
| --- | --- | --- | --- |
| qPCR primers | RpLp0-fwd | AGATTCGGGATATGCTGTTGGC | 109-bp amplicon |
|  | RpLp0-rev | TCGGGTCCTAGACCAGTGTTC | RpLp0 |
| qPCR primers | DAO-fwd-1758 | CTGGTGCACTACCGTGTTGA | 139-bp amplicon |
|  | DAO-rev-2729 | ACTGGGTCTGCTCAAGTGTG | DAO |
|  | fwHDC116 | GAGCCCTGTGAATACCGTGAA | 117-bp amplicon |
|  | rvHDC233 | GCACATTTGGAGTCACCTGC | HDC |
|  | HNMT-548-fwd | CATGGTCTCTTAGCTGCCAGTG | 158-bp amplicon |
|  | HNMT-706-rev | CAGGTCATCCAGTATCTGCGCA | HNMT |
| GT primers | Ef2 6864 | GGTTGCTCGGTGTGTTTCATGGAT | 258-bp amplicon |
|  | Er3 6868 | GCTGTGCTCTGAAACATTTTCTGTGGTC | DAO WT allele |
|  | Ef 6863 | TCAGCGCCTTCTGAGGTTGCTC | 315-bp amplicon |
|  | Kr 3278 | GGGCAAGAACATAAAGTGACCCTCC | DAO KO allele |

RpLp0= Ribosomal Protein Large P0; GT=Genotyping; DAO=Diamine oxidase; HDC=Histidine decarboxylase; HNMT=Histamine N-methyltransferase; fwd=forward; rev=reverse.

Genotyping

The distal phalanx was used for genotyping. Samples were digested overnight at 56°C using 250 µl lysis-buffer (10 mmol/l Tris-Cl pH8, 100 mmol/l NaCl, 1 mmol/l EDTA, 1%SDS, 0.5 mg/ml Proteinase K [P2308, Sigma-Aldrich, Austria]). Proteinase K was inactivated at 70°C for 15 minutes. Samples were diluted 1:100 in ddH2O and used in the PCR reaction.

The GoTaq G2 Hot Start Master Mix (M7422, Promega, USA) was used according to the instructions provided by the manufacturer. Primers stated in Online Resource table 1 were used. PCR products were analyzed using a 3% Agarose gel with PeqGreen (732-3196, VWR, Germany). This multiplex PCR detected the WT allele using Ef2 6864 and Er3 6868 primers, and the KO allele using the Ef 6863 and Kr 3278 primers. Heterozygous animals were positive for both alleles. While the Ef2 6864, Ef 6863 and Kr 3278 primers are between exon 1 and 2 of the murine AOC1 gene, the Kr 3278 primer is solely found on the construct used for AOC1 deletion.

Quantitative PCR of DAO, HNMT and HDC

Expression of DAO (Online Resource Fig. 1a), HNMT (Online Resource Fig. 1b) and HDC (Online Resource Fig. 1c) in different tissues is shown relative to WT-Duodenum samples for DAO and HNMT. No expression of HDC was measured in WT-Duodenum and therefore the WT-stomach samples were used for calculation of relative gene expression. RpLp0 was used as a reference gene. Samples represent the mean relative expression of duplicates.

**Administered substances**

Histamine: Histamine dihydrochloride (H7250, Sigma-Aldrich, Austria) was dissolved in H_2_O and frozen at -30°C. A fresh aliquot was thawed for each experiment and further diluted in saline.

Propranolol: Propranolol hydrochloride (P0884, Sigma-Aldrich, Austria) was freshly dissolved in H_2_O for each experiment and further diluted in saline.

Metoprine: Metoprine (M338835, Toronto Research Chemicals,Canada) was freshly dissolved in 10% lactic acid (L1875, Sigma-Aldrich, Austria) for each experiment and further diluted in saline.

Tacrine: Tetrahydroaminacrin-hydrochloride (A79922, Sigma-Aldrich, Austria) was freshly dissolved in H_2_O for each experiment and further diluted in saline.

Folic acid: Folic acid (F7876, Sigma-Aldrich, Austria) was freshly dissolved in H_2_O for each experiment and further diluted in saline.

DAO: recombinant human DAO was provided by EG in 50 mM Hepes and 150 mM KCl at pH 7.4. Aliquots were stored at -30°C, freshly thawed for each experiment and further diluted in saline.

Tissue expression of DAO, HNMT and HDC


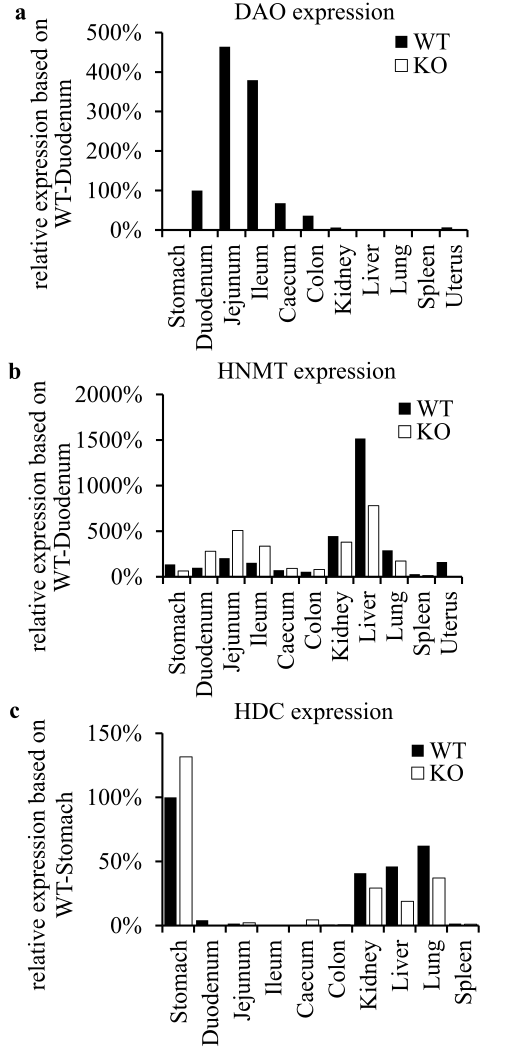


**Online Resource Fig. 1** Relative expression of DAO, HNMT and HDC in different tissues of DAO KO and WT mice

Relative expression is shown as the mean of duplicates. For DAO and HNMT duodenum and for HDC stomach mRNA expression data were used as 100%. Rplp0 mRNA expression was employed as the reference gene for normalization.

The high expression of HDC in the kidneys, liver and lung might be partially responsible for the increased plasma histamine concentrations in rodents compared to dogs, pigs or humans. The liver and kidneys are tissues with low mast cell density and therefore mast cells are unlikely to be responsible for the relatively high HDC mRNA levels.

Local histamine concentrations after mast cell degranulation

Tissue histamine concentrations have been estimated to be between 1 to 100 µg/g or 9 to 900 nmole/g or 9 to 900 µM converting g into ml.[1] Hesterberg et al. (1984) [2] published 11, 31 and 8 µg/g or 99, 279 or 72 µM histamine content for human lung, jejunum/ileum and skin tissue respectively. At the time, the authors were one of the leading groups in the “histamine” field, particularly known for the development of meticulous methods to accurately measure histamine concentrations. The jejunum/ileum and skin store about 50% and 30% respectively of the total histamine in the body.[3] Almost all of this histamine is stored in mast cells and the majority of mast cells are close to vessels. If 25% of mast cells degranulate at the same time and release 40% of their histamine, the interstitial fluid histamine concentrations in lung, jejunum/ileum and skin tissue would increase to 9.9, 28 and 7.2 µM respectively. The steady state histamine concentration without mast cell degranulation is likely negligible. Nevertheless, the interstitial fluid compartment comprises only 15% of the total tissue volume and therefore histamine is “enriched” 6.7-fold to 66, 181 and 48 µM. We are not aware of accurate mice or rat data which would allow us to perform similar calculations. These calculations might be still an underestimation of the histamine concentrations around blood vessels, because it is well known that mast cells localize close to vascular cells.

Calculation of theoretical histamine half-lives based on different glomerular filtration rates (GFRs) in humans and mice

In humans we calculated the theoretical plasma histamine half-life based on a GFR of 100 ml/min and a plasma volume of 3000 ml. We did not include protein binding of histamine in our calculations, because the mean is only 13.4%.[4] Assuming an initial histamine concentration of 100 ng/ml we calculated a histamine half-life of 20, 41 and 104 minutes at a GFR of 100 (100%), 50 (50%) and 25 (25%) ml/min respectively (Online Resource Fig. 2a).

We calculated the theoretical plasma histamine half-life based on a 20 g mouse with a GFR of 0.20 ml/kg/min or 10 µl/g/min, a blood volume of 80 ml/kg and a hematocrit of 40% resulting in 0.96 ml total plasma volume. We did not include protein binding of histamine in our calculations because the mean is only 13.4% (Williams and Shale 1992). Assuming an initial histamine concentration of 100 ng/ml we calculated a histamine half-life of 3, 6.3 and 13 minutes at a GFR of 0.20 (100%), 0.10 (50%) and 0.05 ml/min (25%) respectively (Online Resource Fig. 2b).


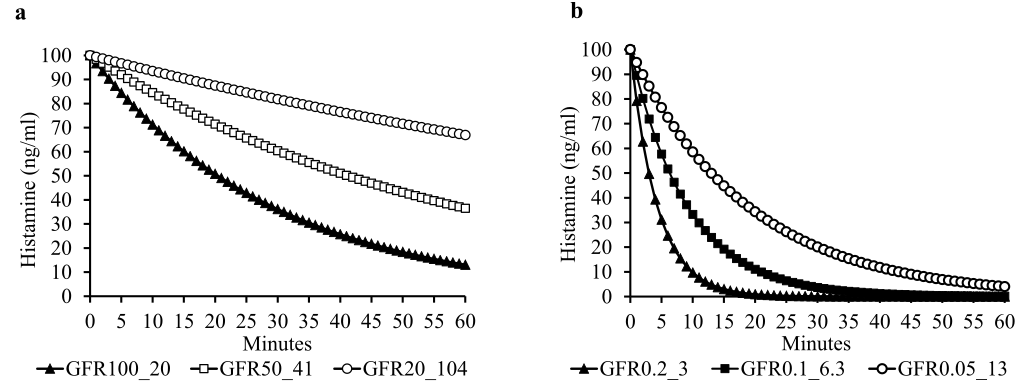


**Online Resource Fig. 2** Calculated histamine half-lives based on different glomerular filtration rates (GFR) in mice and humans

(a) Humans: GFR 100, 50 and 25 ml/min and a plasma volume of 3000 ml. The half-lives are added after the GFR rates. (b) Mice: GFR 0.20, 0.10 and 0.05 ml/min or 10, 5 and 2.5 µl/g/min and a plasma volume of 0.96 ml in a 20 g mouse. The half-lives are added after the GFR rates.

Comparison of mice with and without HNMT blockage in a subcutaneous histamine challenge model


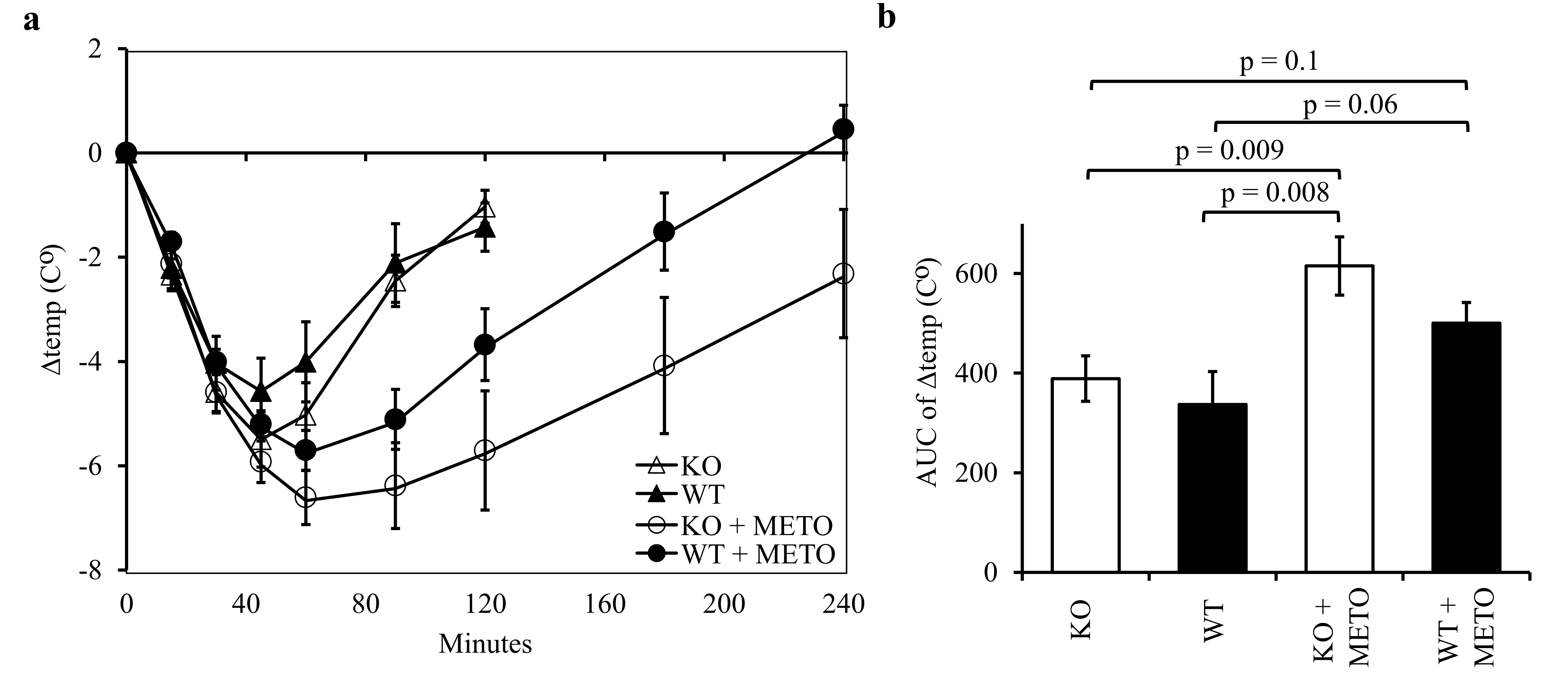


**Online Resource Fig. 3** Inhibition of HNMT prolongs recovery time after histamine challenge in DAO KO mice

(a) All mice were treated with the β-adrenergic blocker propranolol at 2 mg/kg i.p. 20 minutes prior to challenge with subcutaneous (s.c.) histamine (5 mg/kg). WT and DAO KO mice received metoprine (METO) intraperitoneally (3 mg/kg i.p.) 60 minutes before challenge with histamine. Mice receiving metoprine (n = 11 per genotype, ● for WT and ○ for DAO KO) were compared to mice without metoprine pretreatment (n = 9 per genotype, ▲ for WT and ∆ for DAO KO). Differences in core body temperature normalized to baseline are shown as mean ±SEM. The data are also shown in the main text (Fig. 2c and 3d). (b) The area under the curves (AUCs) of the reduction in body temperature to baseline were calculated from 0 to 120 minutes and are shown as mean ±SEM. P-values were calculated using a two-sided t-test of individual AUCs.

Comparison of DAO WT and KO mice with and without acute kidney injury in a subcutaneous histamine challenge model


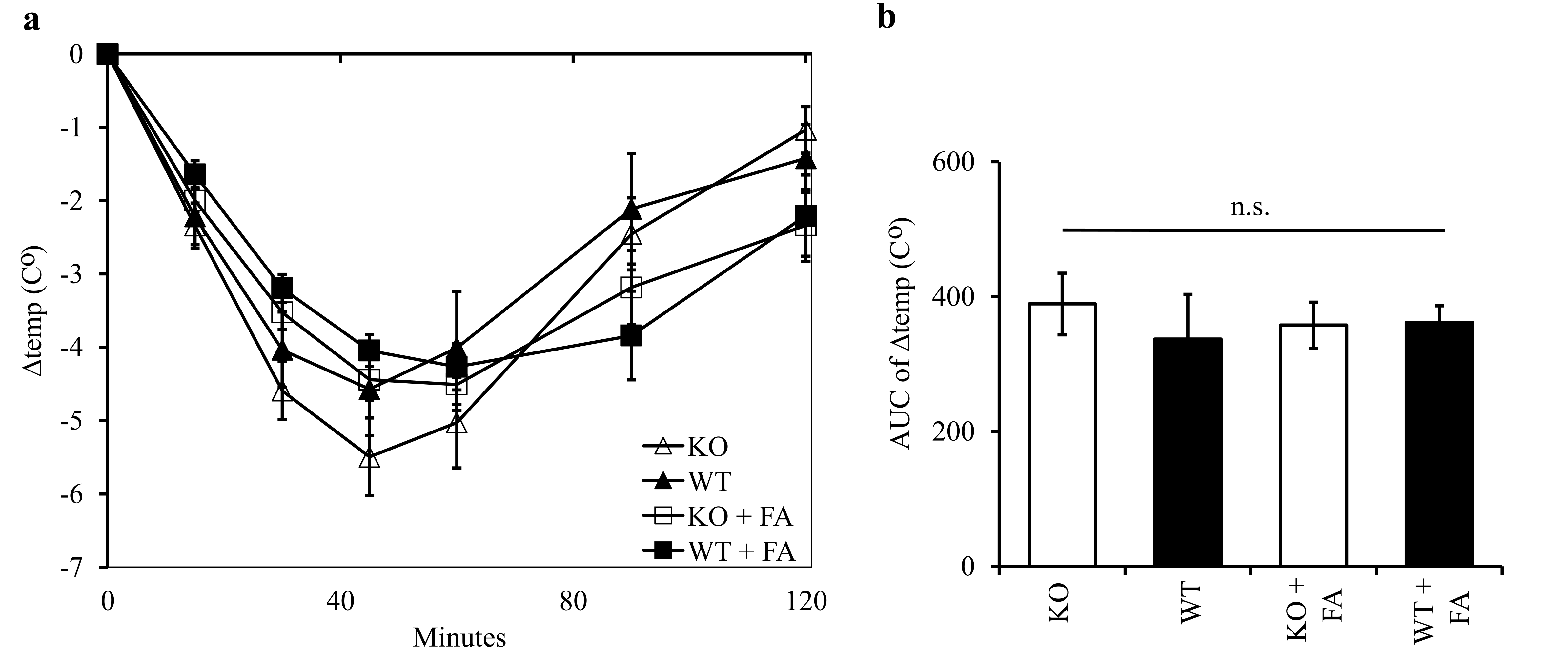


**Online Resource Fig. 4** Acute kidney injury does not change recovery time after histamine challenge in DAO KO and WT mice

(a) All mice were treated with the β-adrenergic blocker propranolol at 2 mg/kg i.p. 20 minutes prior to challenge with subcutaneous (s.c.) histamine (5 mg/kg). WT and DAO KO mice received 100 mg/kg folic acid (FA) intraperitoneal (i.p.) causing acute kidney injury within 48 hours (n = 10 for WT ■ and n = 9 for DAO KO □). These mice were compared to mice without FA pretreatment (n = 9 per genotype, ▲ for WT and ∆ for DAO KO). The data are also shown in the main text (Fig. 2c and 4b). The differences in core body temperature normalized to baseline are shown as mean ±SEM. (b) The area under the curves (AUCs) of the reduction in body temperature compared to baseline were calculated from 0 to 120 minutes and are shown as mean ±SEM. P-values were calculated using a two-sided t‑test of individual AUCs. n.s. = not significant.


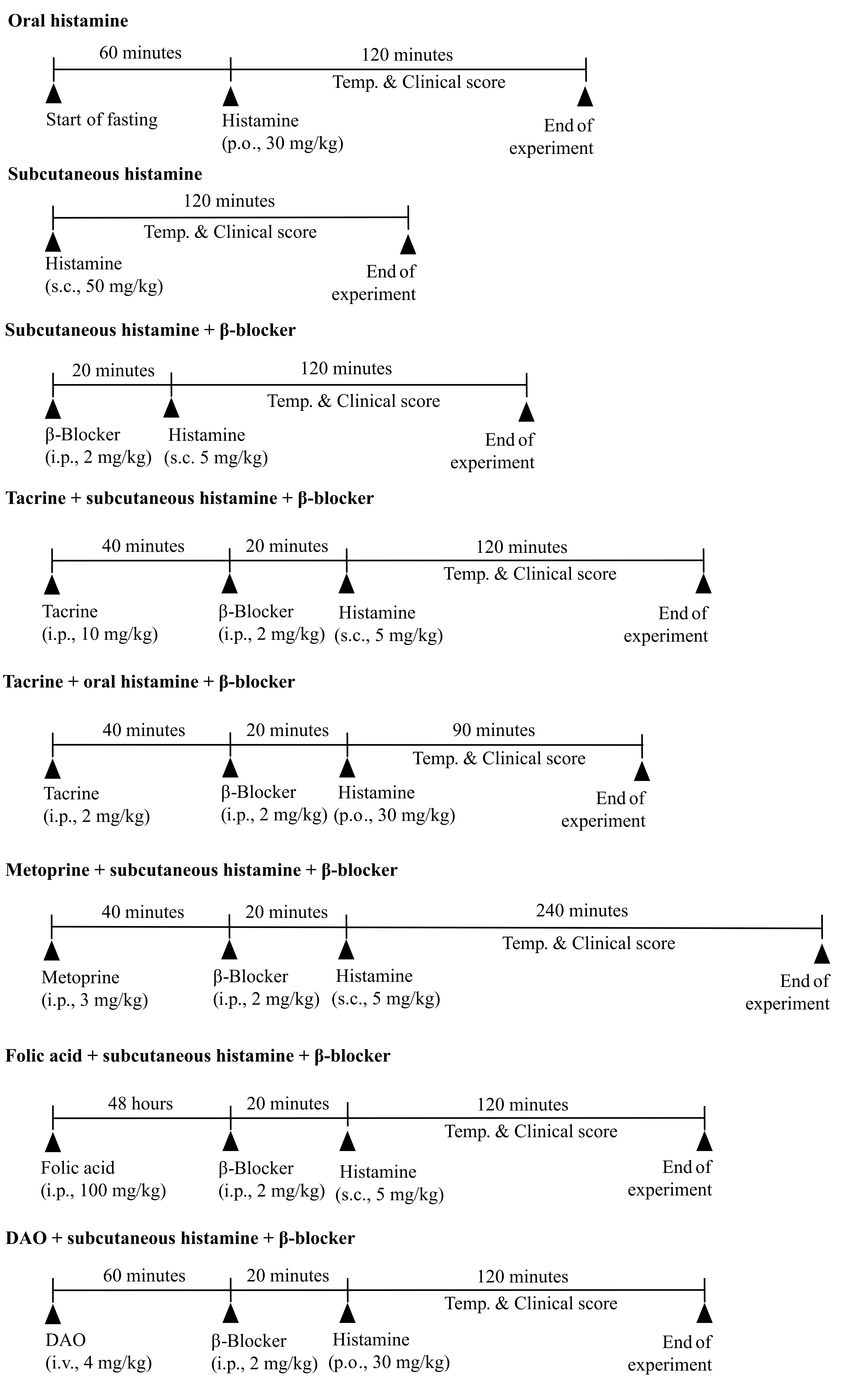


**Online Resource Fig. 5** Dosing scheme for mice in the present study

s.c.: subcutaneous, i.p.: intraperitoneal, i.v.: intravenous, β-blocker: Propranolol, Temp & Clinical score: non-invasive temperature measurement using a previously implanted (IPTT-300, BioMedic Data Systems Inc., USA) subcutaneous microchip and determination of clinical score at minute 0, 15, 30, 45, 60, 90, (120, 180, 240).

**References (Supplement)**

[1] Packard KA, Khan MM. Effects of histamine on Th1/Th2 cytokine balance. Int Immunopharmacol 2003; 3: 909–920. doi:10.1016/S1567-5769(02)00235-7

[2] Hesterberg R, Sattler J, Lorenz W, et al. Histamine content, diamine oxidase activity and histamine methyltransferase activity in human tissues: Fact or fictions? Agents Actions 1984; 14: 325–334. doi:10.1007/BF01973821

[3] Boehm T, Ristl R, Joseph S, et al. Metabolome and lipidome derangements during a severe mast cell activation event in a patient with indolent systemic mastocytosis. J Allergy Clin Immunol 2021; 148: 1533–1544. doi:10.1016/j.jaci.2021.03.043

[4] Williams WR, Shale DJ. In vitro displacement of vasoactive mediators from plasma proteins: a possible mechanism for pseudo-allergic reactions to neuromuscular blocking drugs. Br J Anaesth 1992; 69: 508–510. doi:10.1093/bja/69.5.508
